# Association between bacterial vaginosis with human papillomavirus in the United States (NHANES 2003–2004)

**DOI:** 10.1186/s12905-024-02956-w

**Published:** 2024-02-22

**Authors:** Jie Qi, Congwei Dai, Liyun Song, Junqin Zhang

**Affiliations:** Department of Gynecology, Hebei General Hospital, Shijiazhuang, 050000 China

**Keywords:** Bacterial vaginosis, HPV, NHANES (National Health and Nutrition Examination Survey), Logistic model

## Abstract

**Background:**

The balance of vaginal microecology is closely related to human papillomavirus (HPV) infection and cervical lesions. This study aims to investigate the relationship between bacterial vaginosis (BV) and HPV infection.

**Methods:**

In total, 1,310 individuals from the National Health and Nutrition Examination Survey (NHANES, 2003–2004) were included in this study. Logistic regression and subgroup analyses were used to examine the association between BV and HPV infection.

**Results:**

A significant positive association was observed between BV and HPV infection in women after adjustment for other confounders (OR = 1.47, 95% confidence interval [CI]: 1.15–1.88). In subgroup analyses, we have found this positive correlation was most prominent among Mexican Americans (OR = 1.83, 95% CI: 1.08–3.08) and non-Hispanic blacks (OR = 1.81, 95% CI: 1.08–3.04).

**Conclusions:**

This cross-sectional study demonstrated a positive association between BV and HPV infection in women.

**Supplementary Information:**

The online version contains supplementary material available at 10.1186/s12905-024-02956-w.

## Background

Understanding the relationship and developmental mechanisms linking bacterial vaginosis (BV) and cervical human papillomavirus (HPV) infection is crucial for advancing the elimination of HPV and the treatment of cervical lesions. Moreover, in 2020, the World Health Organization reported an estimated 600,000 new cases of cervical cancer globally [1]. Persistent infection with high-risk HPVs (HR-HPVs) has been well-established to be the cause of cervical cancer. Persistent infection with HR-HPV is considered a necessary factor for cervical cancer development [2]. The introduction of the HPV vaccine can substantially reduce HPV infection rates; however, the pace of vaccination progress has been slow in developing countries [3]. Therefore, one of the most important approaches to stopping further progression of cervical lesions involves facilitating HPV clearance. BV represents the bacterial alteration of the bacterial community within the reproductive tract, typically characterized by the replacement of the dominant *Lactobacillus* with a higher concentration of *Gardnerella vaginalis* (GV) and several potentially pathogenic microorganisms closely associated with BV. Studies have reported that vaginal infections (the most common types are BV, mycotic vaginitis, and *Trichomonas vaginalis* [TV]) are cofactors for cervical cancer [4–6]. According to various studies, vaginal microbiota plays a crucial role in preventing HPV infection and accelerating HPV clearance, and an imbalance in homeostasis may be a synergistic factor for HPV infection [7, 8]. However, the available epidemiological studies have shown conflicting results regarding the association between BV and cervical HPV infection. In this study, we use the National Health and Nutrition Examination Survey (NHANES) database to investigate the association between BV and HPV in U.S. women.

## Methods

All data in this analysis were extracted from the National Health and Nutrition Examination Survey, an ongoing project to assess the overall health and nutrition status of children and adults in the United States. In this study, we obtained nationally representative population-based health outcome data from the 2003–2004 NHANES cycle. Furthermore, BV data were available only in the 2001–2002 and 2003–2004 NHANES cycles, and laboratory data on HPV were available in the 2003–2020 cycle. The design of this study was cross-sectional in nature.

Population-based data for our study were obtained from the 2003–2004 U.S. NHANES, which includes interviews and medical examinations focusing on various health and nutrition measures. All participants provided written informed consent, and the study was approved by the NCHS Research Ethics Review Board. Participants included 1310 women aged 18–49 years who had participated in the 2003–2004 NHANES and had complete Nugent score and HPV test data. Those who did not have HPV testing (*n* = 272) and Nugent scores (*n* = 276) were excluded from the analysis.

The BV score was calculated according to the method of Nugent. The interpretive reporting for BV involved categorizing BV scores from 0 to 3 indicative of normal vaginal flora, 4–6 as intermediate, 7–10 as indicative of BV [9]. A result was defined either as BV confirmed (positive or intermediate) or not (negative).

HPV infection was described using the following detailed measurement procedure: (1): extraction of vaginal cells from participants with vaginal wipes; (2): processed samples were stored and sent to the Centers for Disease Control and Prevention, Atlanta, GA, for analysis; and (3): DNA from the vaginal swab samples was detected and analyzed using the Roche prototype line blot assay and the Roche Linear Array (LA) HPV genotyping kit.HPV infection was recorded for a dichotomous variable (1 = infected with HPV; 0 = not infected with HPV). Samples of cervical smears were tested using Roche Linear Array HPV Genotyping for HPV DNA. For detailed information regarding the HPV testing procedure, reference can be made to the NHANES website(https://wwwn.cdc.gov/nchs/data/nhanes/2003-2004/labmethods/HPVSWC_I_HPVC_I_R_MET.pdf). The following factors associated with HPV infection were used as covariates. Sociodemographic data: age (years), race (1: Mexican American 2: non-Hispanic black, 3: non-Hispanic white,4: Other Hispanic, and 5: other races), education level (< high school,2: high school,3: >high school), marital status (1:married and living with partner 2:living alone), body mass index (BMI, < 25, ≥ 25–30, ≥ 30), household income-to-poverty ratio (PIR). Other health-related variables included smoking (defined as having smoked at least 100 cigarettes in lifetime) and alcohol (never : had < 12 drinks in lifetime; former: had ≥ 12 drinks in 1 year and did not drink last year, or did not drink last year but drank ≥ 12 drinks in lifetime; yes: drink in the last year; Missing: Missing data.), and diabetes(The diagnostic criteria for diabetes are:1.doctor told you have diabetes, 2.glycohemoglobin HbA1c(%) > = 6.5, 3.fasting glucose (mmol/l) > = 7.0, 4.random blood glucose (mmol/l) > = 11.1, 5.Use of diabetes medication or insulin; Prediabetes: Hba1c: >=5.7 and < 6.5,FPG: 5.6-7.0).

### Statistical analysis

All statistical analyses considered the NHANES sample weight. Multivariate logistic regression was used to assess the association between BV and HPV by adjusting for covariates. We evaluated the false discovery rate (FDR) for conducting multiple comparisons such as Benjamini–Hochberg (BH) adjustment in Additional file [10]. Continuous variables were expressed as mean ± standard deviation and categorical variables as percentages. In our study, three models have been developed: the non-adjusted model: no covariates adjusted for; the minimally-adjusted model: adjusted only for age and race; the fully-adjusted model: adjusted for all covariates presented in Table 1. Additionally, subgroup analyses were conducted based on age, race, and BMI. Data analyses were performed using the statistical software packages R (http://www.R-project.org) and Empower (R) (www.empowerstats.com, X&Ysolutions, inc. Boston MA) [11]. We considered a *P* value < 0.05 to indicate statistical significance.

**Table 1:** Baseline characteristics of 1310 participants in the 2003–2004 NHANES database

HPV	Total	Negative	Positive	*P*-value
**N**	1310	650	660	
**Age, year**	30.98 ± 9.91	31.54 ± 9.96	30.43 ± 9.83	0.041
**PIR**	2.24 ± 1.65	2.41 ± 1.67	2.06 ± 1.61	< 0.001
**BMI, kg/m** ^**2**^	28.33 ± 7.50	28.02 ± 7.46	28.64 ± 7.54	0.139
**Race**				< 0.001
Mexican American	292 (22.29%)	162 (24.92%)	130 (19.70%)	
Non-Hispanic Black	345 (26.34%)	127 (19.54%)	218 (33.03%)	
Non-Hispanic White	579 (44.20%)	321 (49.38%)	258 (39.09%)	
Other Hispanic	46 (3.51%)	14 (2.15%)	32 (4.85%)	
Other Race	48 (3.66%)	26 (4.00%)	22 (3.33%)	
**Marital status**				< 0.001
Married and living with partner	653 (49.85%)	366 (56.31%)	287 (43.48%)	
Living alone	657 (50.15%)	284 (43.69%)	373 (56.52%)	
**Education level**				0.004
<high school	70 (5.34%)	36 (5.54%)	34 (5.15%)	
high school	608 (46.41%)	272 (41.85%)	336 (50.91%)	
>high school	632 (48.24%)	342 (52.62%)	290 (43.94%)	
**Drinking**				0.840
Never	151 (11.53%)	78 (12.00%)	73 (11.06%)	
Former	155 (11.83%)	74 (11.38%)	81 (12.27%)	
Now	652 (49.77%)	328 (50.46%)	324 (49.09%)	
Missing	352 (26.87%)	170 (26.15%)	182 (27.58%)	
**Smoking**				0.019
NO	629 (48.02%)	336 (51.69%)	293 (44.39%)	
Yes	425 (32.44%)	190 (29.23%)	235 (35.61%)	
Missing	256 (19.54%)	124 (19.08%)	132 (20.00%)	
**Diabetes**				0.689
No	1097 (86.38%)	545 (85.96%)	552 (86.79%)	
Pre-DM	120 (9.45%)	64 (10.09%)	56 (8.81%)	
yes	53 (4.17%)	25 (3.94%)	28 (4.40%)	
**BV**				< 0.001
Negative	543 (41.45%)	307 (47.23%)	236 (35.76%)	
Positive	767 (58.55%)	343 (52.77%)	424 (64.24%)	

Data in the table:

For continuous variables: *P*-value was by survey-weighted linear regression. For categorical variables: *P*-value was by survey-weighted Chi-square test。BMI, body mass index; NHANES, National Health and Nutrition Examination Survey; SD, standard deviation

## Results

**Fig. 1:**
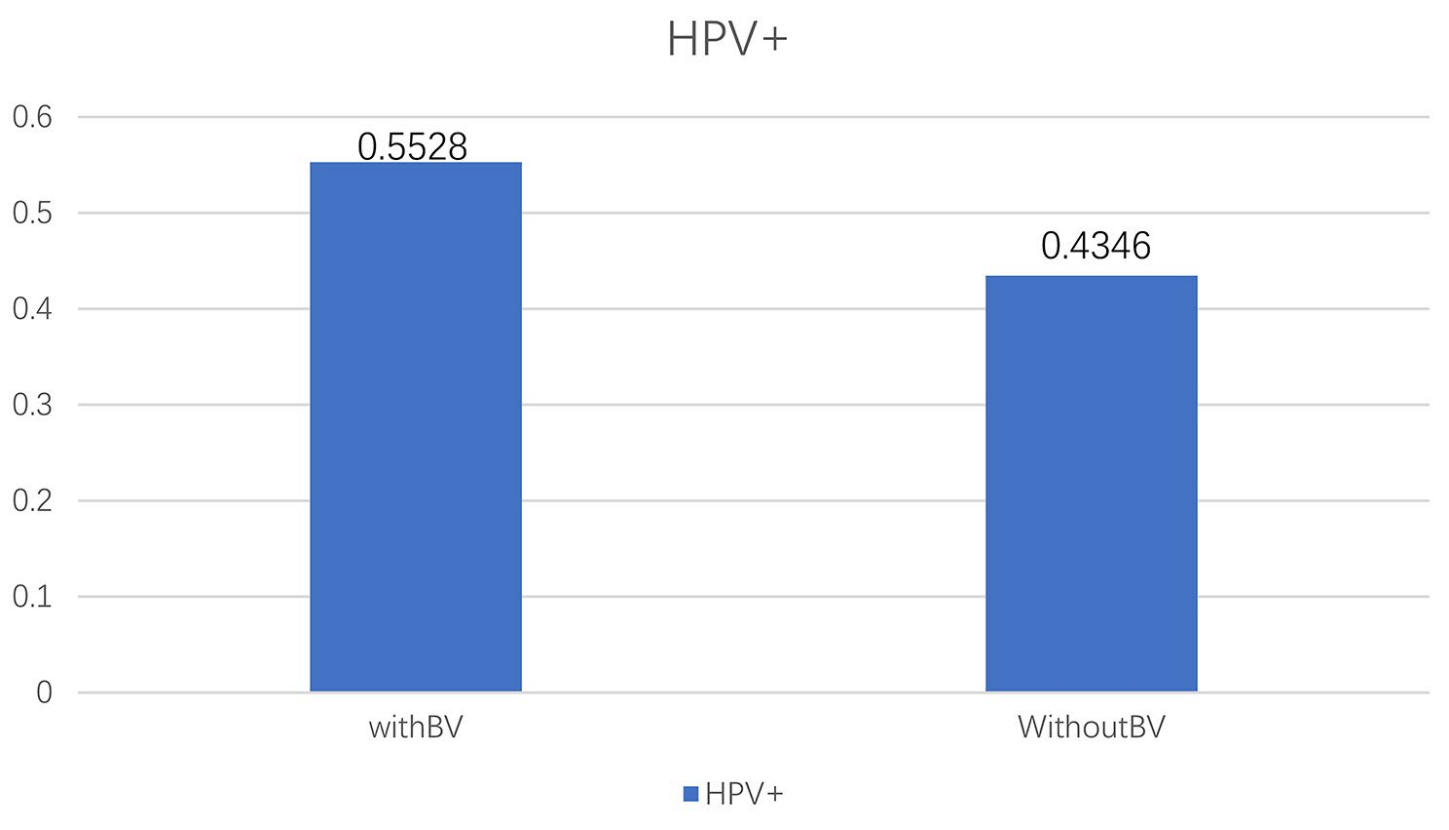
HPV prevalence by BV status

**Fig. 2:**
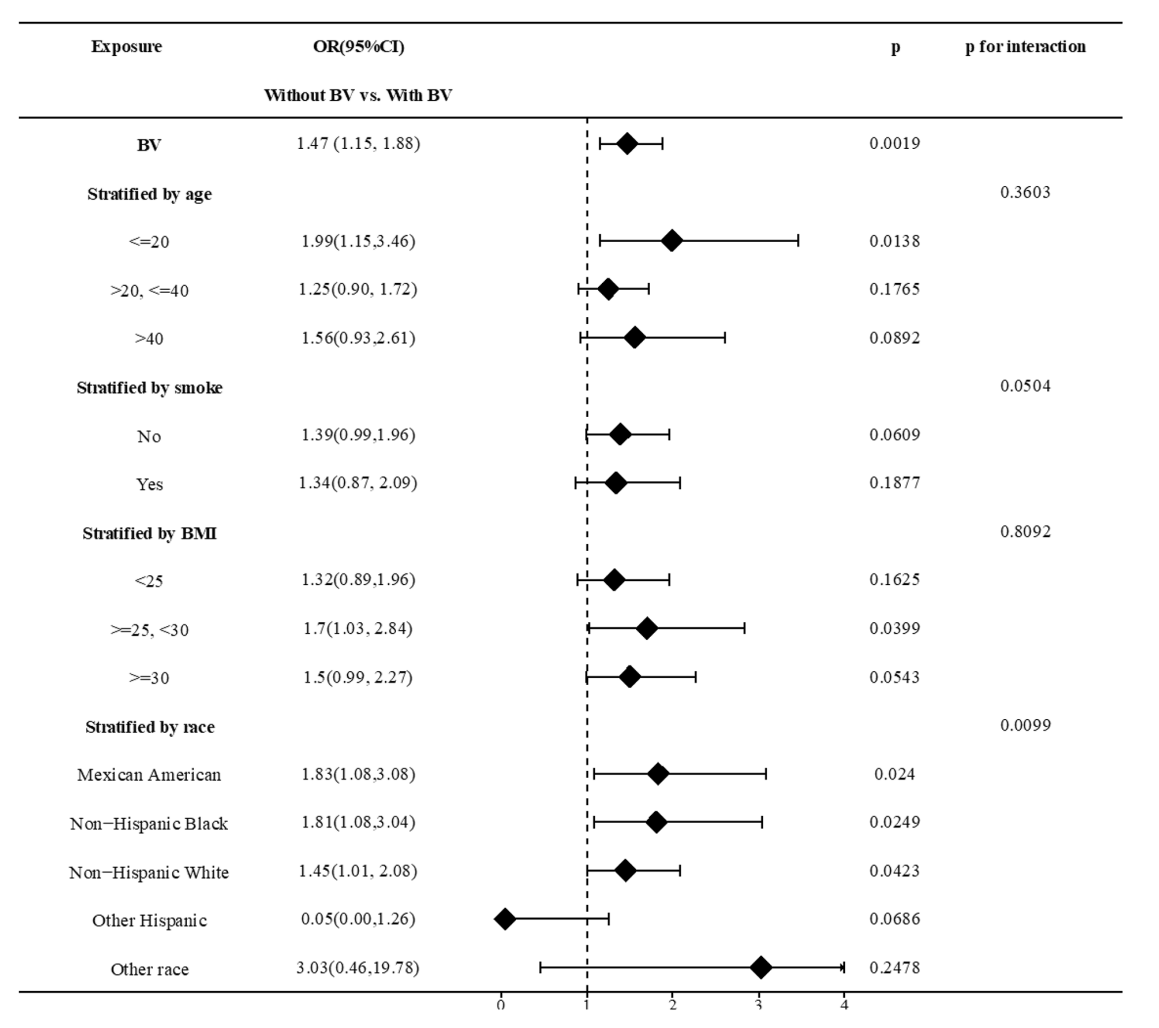
Subgroup analysis for the association between DII and infertility

For this analysis, a total of 1,310 individuals were selected in the years 2003–2004. Of them,50.4% had an HPV infection. Patient characteristics associated with HPV groups are shown in Table 1. Those with HPV infection were younger (30.43 ± 9.83) and more likely to be poorer (2.06 ± 1.61). Moreover, BV was detected in 64.24% in individuals who tested positive for HPV. Figure 1 shows HPV infection rates among women with and without BV. Briefly, women with BV had a higher prevalence rate of HPV (55.28% vs. 44.72%). After constructing three regression models (non-adjusted model: no covariates were adjusted for, minimally-adjusted model: adjusted only for age and race, fully-adjusted model: adjusted for all covariates presented in Table 1), women with BV exhibited a significant association with HPV (model 1:OR = 1.61, 95% CI: 1.29–2.01; model 2:OR = 1.49, 95% CI:1.18–1.87; model 3:OR = 1.47, 95% CI: 1.15–1.88). A stratified analysis was also conducted to explore potential factors that could impact the effect sizes in HPV and BV (Fig. 2). Our subgroup analyses revealed that race affected the effect size of the relationship (p for interaction: 0.0099), and this positive correlation was slightly stronger in Mexican Americans (OR = 1.83, 95% CI: 1.08–3.08) as well as non-Hispanic black individuals (OR = 1.81, 95% CI: 1.08–3.04) compared to non-Hispanic Whites (OR = 1.45, 95% CI: 1.01–2.08).

## Discussion

The maintenance of a balanced vaginal microbiota is crucial for preventing infections of the female genital tract and minimizing the risk of cervical lesions [12]. In the context of vaginal microecological instability, the presence of M. mulieris and Gardnerella species increased the incidence of BV, and the presence of C. albicans increased the incidence of vulvovaginal candidiasis (VC) in females [10]. David Pacha-Herrera et al. report probiotic activity of Lactobacillus in the female vagina [13]. Once the balance is disturbed, the immune system of the vagina is compromised, making it more susceptible to inflammation [14]. The occurrence of inflammation can damage the integrity of the cervical epithelium, creating opportunities for HPV to penetrate the basement membrane and cause infection. Furthermore, nonspecific antibacterial oxidants may be produced, potentially causing DNA damage in the host [15]. Our findings revealed that HPV infection was positively correlated with BV. Additionally, this correlation is the strongest correlation in Mexican American individuals (OR = 1.83, 95% CI: 1.08– 3.08). Consistent with our analysis, a previous systematic review [16] found a positive association between BV and cervical HPV infection (OR 2.62, 95% CI 1.84–3.73, *P* < 0.05). However, the study by Verteramo et al. [9] showed that bacterial vaginosis did not correlate with HPV infection. Heterogeneity between studies, including confounding factors such as study design, sample, and control, may explain the different results. Our results suggest that bacterial vaginosis is associated with HPV infection and BV may have a synergistic effect with HPV infection.

Cervical mucosal immunity comprises innate and adaptive immunity, with the microbial barrier being an essential component of primary immunity [17]. The predominant species in healthy women of childbearing age is *Lactobacillus*, regardless of ethnic differences in vaginal microbiota [18, 19]. However, BV may alter the cervicovaginal microenvironment from the dominant *Lactobacilli* to a dysbiosis composed of strictly facultative anaerobic bacteria [20]. BV also induces an inflammatory response in the epithelial cells of the cervix [21, 22]. Co-culturing BV-associated pathogens including *Gardnerella*, *Prevotella*, *Atopobium vaginae, Sneathia amnii*, with a representative health-associated commensal *Lactobacillus crispatus* using a 3D cell model of the cervix revealed that four pathogens induced the production of multiple pro-inflammatory molecules [23]. BRISELDEN found that 84% of women with BV were sialidase positive [24]. BV-related sialidase-positive bacteria can break down mucus and damage the cervical epithelial tissue, producing biogenic amines and causing oxidative stress, thus increasing the risk of HPV persistent infection and lesion development [12]. Conversely, *Lactobacillus* can release antibacterial peptides, bacteriocins, hydrogen peroxide, and surfactants to reduce the synthesis of oncogenic substances and inhibit the proliferation of malignant cells [25–27]. BV may also affect the immunologic balance in cervical tissue by activating the pro-inflammatory transcription factor NF-κB in cervicovaginal epithelial cells, triggering abundant inflammation and innate immune responses [27]. A clinical trial involving 60 women of reproductive age in the United States and Africa explored the relationship between BV, HPV, and CIN progression. The results showed that an increased BV-related IL-1β/IP-10 cytokine ratio was negatively correlated with clearance of high-risk HPV. Additionally, an increased TNF-α/MIP-1β ratio, another inflammatory feature of BV, was associated with persistent HPV infection and progression to CIN2+ [28]. In summary, the mechanism underlying HPV infection caused by BV is believed to involve a decrease in physical barrier function and local immune dysfunction. Our findings and previous research suggest that BV is associated with an increased risk of cervical HPV infection, and this association may be influenced by ethnicity. Further research is required to elucidate the complex interactions between BV and HPV infection and to identify potential interventions to reduce the risk of cervical lesions.

The greatest strength of this study is that it includes a representative sample from a multiracial population. Furthermore, subgroup analysis was conducted. The weakness of this study is that it had a cross-sectional design, and data on BV and HPV infection were collected simultaneously rather than over time. Thus, the sequential occurrence of HPV and BV infections remains unclear, and whether BV increases the risk of HPV infection or HPV increases susceptibility to BV remains unanswered. This conclusion requires further prospective studies of intervention trials for clarity. Additionally, NHANES only collected data on BV and HPV infection in one cycle, therefore the sample size was relatively small.

Overall, this cross-sectional study shows a positive association between BV and HPV infection, particularly among Mexican Americans and non-Hispanic blacks. Elucidating the occurrence of BV in patients with HPV infection may increase the likelihood of HPV clearance and reduce the possibility of persistent infection, which is important for preventing and treating cervical lesions.

### Electronic supplementary material

Below is the link to the electronic supplementary material.

Supplementary Material 1

Supplementary Material 2

## Data Availability

The datasets generated and analyzed during the current study are available in the repository, which is publicly available at https://www.cdc.gov/nchs/nhanes/index.htm.

## Abbreviations

BMI: Body mass index
BV: Bacterial vaginosis
CI: Confidence interval
GV: *Gardnerella vaginalis*
HPV: Human papillomavirus
HR-HPV: High-risk human papillomavirus
NHANES: National Health and Nutrition Examination Survey
PIR: Income-to-poverty ratio
TV: *Trichomonas vaginalis*
