# Supplementary material for: Association between bacterial vaginosis with human papillomavirus in the United States (NHANES 2003–2004)
Source: BMC Womens Health. 2024 Feb 22;24:138. doi: 10.1186/s12905-024-02956-w (PMC10882805; doi:10.1186/s12905-024-02956-w)
Supplement: Supplementary file 1 — Supplementary Material 1 [file 12905_2024_2956_MOESM1_ESM.docx]

TABLE . Baseline characteristics of 1310 participants in the 2003-2004 NHANES database

|  | **low-risk** | **high-risk** | **Pvalue** |
| --- | --- | --- | --- |
| **Age, year** | 35.08±0.57 | 31.97±0.59 | < 0.001 |
| **PIR** | 2.48±0.17 | 2.42±0.10 | < 0.001 |
| **BMI, kg/m2** | 28.25±0.47 | 28.48±0.50 | 0.17 |
| **Race** |  |  | 0.002 |
| Mexican American | 62(9.74) | 68(8.66) |  |
| Non-Hispanic Black | 92(19.23) | 126(18.51) |  |
| Non-Hispanic White | 109(60.16) | 149(61.72) |  |
| Other Hispanic | 17(8.06) | 15(4.69) |  |
| Other Race | 5(2.81) | 17(6.43) |  |
| **Marital Status** |  |  | < 0.0001 |
| Married and living with partner | 147(56.73) | 140(44.43) |  |
| Living alone | 138(43.27) | 235(55.57) |  |
| **Education** |  |  | 0.14 |
| <high school | 17(2.66) | 17(3.43) |  |
| high school | 140(43.49) | 196(39.85) |  |
| >high school | 128(53.85) | 162(56.72) |  |
| **Drinking** |  |  | 0.08 |
| never | 36(10.61) | 37(10.25) |  |
| former | 36(14.31) | 45(14.59) |  |
| mild | 47(19.68) | 38(17.61) |  |
| moderate | 59(29.20) | 49(20.95) |  |
| heavy | 49(26.19) | 82(36.59) |  |
| **Smoke** |  |  | 0.01 |
| never | 149(54.30) | 144(48.96) |  |
| former | 35(14.34) | 37(11.83) |  |
| now | 63(31.36) | 100(39.21) |  |
| **Diabetes** |  |  | 0.49 |
| No | 239(85.50) | 313(86.83) |  |
| pre-DM | 26( 9.31) | 30( 8.47) |  |
| yes | 12(5.19) | 16(4.70) |  |
| **BV** |  |  | 0.07 |
| negative | 105(43.11) | 131(38.08) |  |
| positive | 180(56.89) | 244(61.92) |  |

Positive for any of the HPV genotypes (HPV16, 18, 31, 33, 35, 39, 45, 51, 52, 56, 58, 59，66，and 68) was regarded as a high-risk HPV infection, positive for the rest of the HPV genotypes was considered as low-risk HPV infection.
