# Supplementary material for: Association between bacterial vaginosis with human papillomavirus in the United States (NHANES 2003–2004)
Source: BMC Womens Health. 2024 Feb 22;24:138. doi: 10.1186/s12905-024-02956-w (PMC10882805; doi:10.1186/s12905-024-02956-w)
Supplement: Supplementary file 2 — Supplementary Material 2 [file 12905_2024_2956_MOESM2_ESM.docx]

Table:Subgroup analyses by stratified logistic regression model of 1310 participants

|  | **Fully-adjusted model** |  |
| --- | --- | --- |
| **Exposure** | **OR,95%CI, P** | **Adjusted** *P***-value** |
|  | **Without BV vs. With BV** |  |
| **BV** | 1.47 (1.15, 1.88) 0.0019 | 0.0023 |
| **Stratifiedby age** |  |  |
| <=20 | 1.99(1.15,3.46) 0.0138 | 0.0142 |
| >20, <=40 | 1.25(0.90, 1.72) 0.1765 | 0.1765 |
| >40 | 1.56(0.93,2.61) 0.0892 | 0.0915 |
| **Stratifiedby smoke** |  |  |
| No | 1.39(0.99,1.96) 0.0609 | 0.0701 |
| Yes | 1.34(0.87, 2.09) 0.1877 | 0.1877 |
| **Stratifiedby BMI** |  |  |
| <25 | 1.32(0.89,1.96) 0.1625 | 0.1723 |
| >=25, <30 | 1.7(1.03, 2.84) 0.0399 | 0.0455 |
| >=30 | 1.5(0.99, 2.27) 0.0543 | 0.0621 |
| **Stratifiedby race** |  |  |
| Mexican American | 1.83(1.08,3.08) 0.024 | 0.0351 |
| Non-Hispanic Black | 1.81(1.08,3.04) 0.0249 | 0.0321 |
| Non-Hispanic White | 1.45(1.01, 2.08) 0.0423 | 0.0534 |
| Other Hispanic | 0.05(0.00,1.26) 0.0686 | 0.0686 |
| Other race | 3.03(0.46,19.78) 0.2478 | 0.3244 |

These P-values evaluated by BH adjustment were illustrated in the table as adjusted P-values.
